# The Risk Factors of High Blood Pressure among Young Adults in the Tujia-Nationality Settlement of China

**DOI:** 10.1155/2017/8315603

**Published:** 2017-08-28

**Authors:** Xiaoli Liu, Zheng Xiang, Xiangrong Shi, Hannah Schenck, Xinfeng Yi, Rong Ni, Chaoneng Liu

**Affiliations:** ^1^PE Department of Hubei University for Nationalities, Enshi, Hubei, China; ^2^Institute of Cardiovascular & Metabolic Disease, University of North Texas Health Science Center, Fort Worth, TX, USA; ^3^National Stadium of Enshi Autonomous Prefecture, Enshi, Hubei, China; ^4^The Central Hospital of Enshi Autonomous Prefecture, Enshi, Hubei, China

## Abstract

Demographics questionnaires, and fitness tests were utilized to identify the risk factors of hypertension among younger adults in the years 2005, 2010, and 2014 in China's southwest province of Hubei. The results demonstrated that the prevalence of hypertension was higher between 2011 and 2014 among the young people in this area. The main risk factors of developing hypertension were found to be sex (as man), individuals over 40 years old, blue collar employees who worked in rural areas, overweight/obesity, and those with the low CRF.

## 1. Background

Hypertension is associated with increased risks of stroke, ischemic heart disease [1], heart failure, kidney disease [2], and premature mortality [3]. In China, the prevalence of hypertension among adults aged 18 years and older is substantial [4]. While more common in the older population, an increasing incidence in the younger population is being observed [5]. However, young adults meeting hypertension diagnostic criteria have a lower prevalence of a hypertension diagnosis than middle-aged and older adults [6]. So identifying young individuals who present with a greater risk for developing hypertension may help target public health prevention efforts.

The Tujia-Nationality settlement is the most poverty stricken area in the southwest Hubei province of China, though the implementation of highway and railway systems has rapidly improved the economy from 2011 to 2014. In 2005, the average salary of white collar and blue collar workers in urban areas was $1910 and $1860 per year, compared to $1260 per year that was earned by blue collar workers in rural areas. In 2010, the average salary of white collar workers and blue collar workers in urban areas was $4180 and $3480 per year, compared to $2430 per year that was earned by blue collar workers in rural areas. Finally, in 2014, the average salary of white collar and blue collar workers in urban areas increased to $7000 and $5900 per year, respectively, compared to the $3300 per year that was earned by blue collar workers in rural areas. Young people encounter intense pressure regarding any changes in the economy, which is a main factor of many chronic diseases. However, to our knowledge, few studies focus on the changes of chronic diseases on the young people in this area, particularly hypertension. Our study analyzed blood pressure data in the years 2005, 2010, and 2014 to describe the predominant risk factors associated with hypertension within this area's younger population.

## 2. Material and Methods

### 2.1. Study Population

This study consisted of 3 cross-sectional surveys in the years 2005, 2010, and 2014, and it was designed to investigate the risk factors of hypertension in the southwest Hubei province of China. A total of 4,120 20–44-year-old young adults (age = 31.8 ± 7.2 years) men and women (*n* = 1081 in 2005, *n* = 1461 in 2010, and *n* = 1578 in 2014) participated in this study. All participants were from the same Tujia settlement area in Hubei province of China.

### 2.2. Methods

The testing was comprised of two parts: a demographics questionnaire and a comprehensive physical fitness evaluation. Questionnaires inquired about participants' age, nationality, gender, urban versus rural location, employment classification, and the highest level of completed education. Fitness testing included measurements for blood pressure, height, weight, vital-lung capacity, sidestep test, standing vertical jump, sit-and-reach, grip strength, single leg stance test, reaction time, push-ups (men), and sit-ups (women).

All testing were performed in a gymnasium in July and August of the testing year (2005, 2010, and 2014). The study subjects first completed a questionnaire. Then following a 20 min rest, trained physicians measured subjects' blood pressure using a mercury sphygmomanometer. The measurements were made in triplicate within 10 min interval with the subjects in the seated position. Arterial hypertension was defined as systolic blood pressure ≥ 140 mmHg or diastolic ≥ 90 mmHg or current treatment with antihypertensive drugs.

The cardiorespiratory fitness (CRF) was tested using the sidestep test (TDK-2 intelligent apparatus, Ningbo Jingbei Tiandikuan Electronic Product Manufacturer, China). Man subjects used a 40 cm high footstep and woman subjects used a 35 cm high footstep to perform the up-and-down movement. Every subject performed up-and-down movements for about 3 minutes (90 repetitions) in rhythm to music. After the subject sat down, a finger clip was placed on the subject's middle finger from which pulse rate was displayed on the screen and recorded. Pulse rate readings were taken 3 separate times after the completion of the sidestep exercise: between 1 minute to 1 minute 30 seconds; 2 minutes to 2 minutes 30 seconds; and 3 minutes 30 seconds. The sidestep test index was calculated from the duration of up-and-down movements (in seconds) multiplied by 100 divided by the sum of 3 pulse rate readings.  Table 1 shows the classification criteria of the index as CRF according to the Citizen Physical Health Standard established by Ministry of Education of China and the General Administration of Sport of China [7].

Next we measured the subjects' height, weight, vital-lung capacity, sidestep test, standing vertical jump, sit-and-reach, grip strength, single leg stance test, reaction time, push-ups (male), and sit-ups (female). All of the testing apparatuses were made by the Shenzheng Hengkang Jiaye Limited Company, China. When the subjects were ready, the testers pressed the button to initiate the beginning of the test.

All physical fitness measurements and scoring criteria were classified according to the Citizen Physical Health Standard established by Ministry of Education of China and the General Administration of Sport of China [7]. Each category we measured had an acceptable value range; if the total of these results fell within this range they were labeled as “pass,” while those that did not were labeled as “fail” [7].

Body mass index (BMI) was calculated from weight (in kilograms)/height^2^ (in meter). The BMI was classified into four levels: <25.0 as normal weight, 25.0~29.9 as overweight, and ≥30.0 as obesity.

### 2.3. Statistical Analysis

All statistical analyses were performed using the SPSS for Windows software package (version 18; SPSS Inc., Chicago, IL, USA). Descriptive statistics were obtained first and categorical variables were presented as the number of people (%). Collinearity diagnostics were performed prior to further statistical analysis. A binary unconditional logistic regression model was used for our analysis of the independent effects of each variable. Potential risk factors included testing time, age, nationality, gender, urban versus rural location, employment classification, education level, BMI, CRF, and physical test scores. The dependent factor was “whether the subject is hypertensive” and retained methods used a forward step-by-step approach. All variables significant at *P* < 0.05 were reserved in the final model.

## 3. Result

### 3.1. The Changes of Prevalence of Hypertension, BMI, and CRF from 2005 to 2014

**Figure 1:**
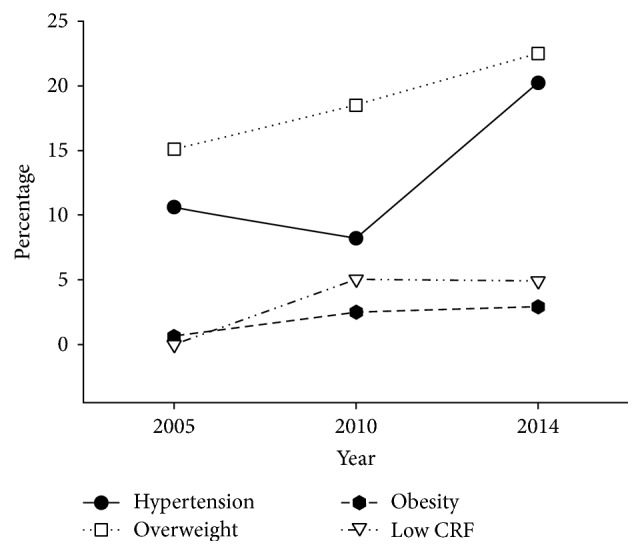
The changes of prevalence of hypertension, BMI, and CRF from 2005 to 2014. BMI = body mass index and CRF = cardiorespiratory fitness.

Of the 4120 participants, 554 of the 4120 young adults were classified as hypertensive, giving an overall prevalence of hypertension was 13.4%. From Figure 1, the prevalence of hypertension was lower at 10.6% in 2005 and 8.2% in 2010 before doubling to 20.2% by 2014. At the same time, the incidence of overweight and obesity was 15.1% and 0.6% in 2005, and they reached 18.5% and 2.5% in 2010 and 22.5% and 2.9% in 2014, respectively. However, the percentages of high CRF, median CRF, and low CRF were 50.6%, 49.4%, and 0% in 2005, and they reached 51.4%, 43.6%, and 5% in 2010, respectively; they were 47.5%, 47.7%, and 4.9% in 2014, respectively.

### 3.2. Prevalence and Distribution of Hypertension

There were differences in the occurrence of hypertension among different age groups, with the likelihood increasing exponentially with age: 8.9% among 20–24-year-olds, 10.2% among 25–29-year-olds, 12.3% in 30–34-year-olds, 14.0% among 35–39-year-olds, and 22.2% among 40–44-year-olds. The prevalence was higher among men at 17.6% compared to women at 9.4% and higher in rural residents at 15.3%, compared to urban residents at 12.6%.

In regard to occupation, the frequency of hypertension was highest among white collar workers at 18.0% and lowest among blue collar workers in urban areas at 11.3%. By level of highest schooling attained, hypertension was highest among participants with a primary school or a middle school education at 15.6%, followed by those with high school education at 13.3%, and university/professional education at 10.9%. Hypertension was also higher in participants with a higher BMI (42.2% of those classified as obesity displayed hypertension). By CRF levels, prevalence was highest among participants who had the low fitness of 25.2%, followed by median fitness of 15.1% and high fitness of 10.5%. Hypertension was higher among individuals who failed the physical test at 15.6% compared to those who passed the test at 12.3%. The distribution of hypertension by the various participant characteristics is summarized in Table 2.

### 3.3. Risk Factors Associated with Hypertension in Binary Logistic Regression Analysis

Table 3 shows the five risk factors associated with developing hypertension. The overall risk of hypertension was the highest in the year 2014; when compared to the same-aged individuals, this risk stood at 49.9% in 2005 and 68% in 2010. The risk of hypertension increased with the age. Compared to the 40–44 age group, the risk of hypertension decreased 58.9%, 59.7%, 51.0%, and 44.6% in 20–24, 25–29, 30–34, and 35–39 age groups, respectively. Men displayed a 49.5% higher risk of hypertension compared to women. Blue collar workers in rural areas had a 1.739-fold (95% CI, 1.258–2.403; *P* = 0.001) risk of hypertension compared to blue collar workers in urban areas. CRF was inversely related to hypertension; those with the low level of CRF had a 2.914-fold risk of hypertension (95% CI, 1.877–4.526; *P* = 0.001), and people with a low level of CRF had a 1.238-fold risk of hypertension (95% CI, 1.004–1.528; *P* = 0.046). Weight, however, was directly related to hypertension; the obesity and overweight had a 6.265-fold and 2.325-fold risk of hypertension compared to normal weight individuals.

## 4. Discussion

This study indicated that the prevalence of hypertension among young adults within the Tujia settlement suddenly increased from 2011 to 2014. Remarkably, just 6.5% of the hypertensive individuals were conscious of their chronic disease. For these young adults the main risk factors of developing hypertension were found to be sex (as man), blue collar workers in rural areas, age over 40 years old, overweight/obesity, and those with low CRF.

We found the prevalence of hypertension suddenly increased from 8.2% in 2010 to 20.2% in 2014, which was even higher than the 6.8% found within the same American age group between 2011 and 2012 [8]. It was a special time for this area in 2010–2014, because the highway and train line had their first routes in 2009 and 2010, leading to rapid development of the local economy. However, healthy lifestyles and health education did not progress at the same pace. The convenience of transport and the development of the Internet made more people opt for a sedentary life [9]. Excessive intake of fat foods led to excess calories and obesity [10]. The CRF, overweight, and obesity increased from 2005 to 2014 (Figure 1), which led to the increase of chronic disease. What made the problem even worse was that just 6.5% hypertensive people knew they had hypertension. So the people in this place must have regular physical examination and necessary health education.

Men have greater levels of oxidative stress than women [11], so they are thus more likely to have oxidative stress-induced increases in their blood pressure [12]. In our study among young adults, men had a higher risk of hypertension, which was consistent with other researches [13]. Because the targeting people were only young adults in this study, women in this area had a higher risk of hypertension once they reached middle age [14].

Since the 1980s, work related stressors have become recognized as significant elements for developing cardiovascular disease and hypertension [15]. Lower socioeconomic status workers (variously defined by education, income, or occupation) have been found to have higher age-adjusted mean systolic blood pressure (by 2-3 mm Hg) or prevalence of hypertension than employees in higher socioeconomic status groups [16]. Our data found blue collar workers in rural areas had a 1.739-fold (95% CI, 1.258–2.403; *P* = 0.001) risk of hypertension compared to blue collar workers in urban areas. Because of the lack of health knowledge and medical care, blue collar workers in rural areas are unaware or lack the resources to prevent chronic disease and form healthier, more sustainable lifestyles [17].

Our study found that those in the 40–44 age group people had the highest risk of hypertension. As the human body ages it becomes less responsive to stimuli, thus making older persons display less sympathetic nervous system upregulation than is seen in younger individuals [17]. Our study demonstrated an age of 40 years was the threshold of the higher hypertensive risk.

The worldwide epidemic of excess body weight including obesity is associated with the increased prevalence of cardiovascular dangers. Obesity is linked to increased sympathetic nervous system activation and increased renin release, which are thought to contribute to hypertension development in obese individuals [18]. Our data found the obese and overweight people had 6.265-fold and 2.325-fold risk of hypertension compared normal weight people. Because obesity is the main risk factor of developing hypertension, maintaining a standard weight is the most effective way to prevent hypertension for young adults in this area.

A higher level of CRF significantly attenuated the rise in blood pressure over the lifespan. Thus, the examination of serial changes in CRF as it relates to incidental hypertension is of considerable interest [19]. Recent findings from animal studies suggest aerobic exercise may prevent increases in blood pressure through beneficial alterations in insulin sensitivity and autonomic nervous system function [20]. Several previous studies have reported the associations between changes in CRF over time and the risk of developing future hypertension. In a Finnish cohort of 163 high-risk males, fitness was inversely associated with carotid intimal-media thickness [21]. Further, the Kuopio Ischemic Heart Disease Risk Factor Study evaluated 854 men with baseline VO_2_ max testing and found that fitness was associated with slower progression of carotid atherosclerosis by high-resolution B-mode ultrasonography at baseline and at an average of 4.2 years later [22]. Our results found the increase in CRF caused a decrease in the risk of hypertension. The hypertension was higher among people who failed the physical test at 15.6% compared to the ones who passed the test at 12.3%. However, it was not included as the risk factor in the logistic model, suggesting that the CRF or aerobic fitness was a more important predictor than the physical fitness in preventing hypertension.

In the present study, we were unable to find a correlation between hypertension and some of the factors that have often been associated with hypertension, such as education [23] and nationalities [24]. This indicates that there must be other risk factors of fundamental importance for hypertension in this area, other than those analyzed here, such as BMI, CRF, employment classification, and sex.

## 5. Conclusion

The prevalence of hypertension among the young Tujia population was higher during the years of 2011–2014. The main risk factors for developing chronic elevated blood pressure were found to be sex (as man), blue collar workers in rural areas, overweight/obesity, individuals over 40 years of age, and those with low CRF.

## Figures and Tables

**Table 1:** Classification criteria of cardiorespiratory fitness (CRF).

Age (yrs)	Low		Median		High	
	Female	Male	Female	Male	Female	Male
20–24	≤46.1	≤46.1	≥46.2, ≤58.0	≥46.2, ≤58.0	≥58.1	≥58.1
25–29	≤46.8	≤46.1	≥46.9, ≤59.1	≥46.2, ≤58.3	≥59.2	≥58.4
30–34	≤47.0	≤46.1	≥47.1, ≤59.9	≥46.2, ≤58.3	≥60.0	≥58.4
35–39	≤46.8	≤46.1	≥46.9, ≤60.3	≥46.2, ≤58.7	≥60.4	≥58.8
40–44	≤46.8	≤46.5	≥46.9, ≤61.5	≥46.6, ≤59.9	≥61.6	≥60.0

**Table 2:** Demographics characteristics of young adults in Tujia-Nationality settlement in China, *N* = 4120.

Variable/category	Normal (%)	Hypertension (%)	Total
Awareness	4084 (99.1)	36 (0.9)	4120
Test time (yr)			
2005	966 (89.4)	115 (10.6)	1081
2010	1341 (91.8)	120 (8.2)	1461
2014	1259 (79.8)	319 (20.2)	1578
Age (yrs)			
20–24	762 (91.1)	74 (8.9)	836
25–29	769 (89.8)	87 (10.2)	836
30–34	733 (87.7)	103 (12.3)	836
35–39	669 (86.0)	109 (14.0)	778
40–44	633 (77.8)	181 (22.2)	814
Nationality			
Han	2115 (85.5)	358 (14.5)	2473
Tujia	1451 (88.1)	196 (11.9)	1647
Sex			
Males	1673 (82.4)	358 (17.6)	2031
Females	1893 (90.6)	196 (9.4)	2089
Urban-rural			
Rural	1116 (84.7)	202 (15.3)	1318
Urban	2450 (87.4)	352 (12.6)	2802
Employment classification			
Blue collar workers in rural area	1116 (84.7)	202 (15.3)	1318
Blue collar workers in urban area	2025 (88.7)	259 (11.3)	2284
White collar workers	425 (82.0)	93 (18.0)	518
Education			
Primary school	313 (84.4)	58 (15.6)	371
Middle school	1078 (84.4)	199 (15.6)	1277
High school	1000 (86.7)	153 (13.3)	1153
College	1175 (89.1)	144 (10.9)	1319
BMI^a^			
Normal weight	2910 (89.9)	326 (10.1)	3236
Overweight	599 (76.0)	189 (24.0)	788
Obesity	52 (57.8)	38 (42.2)	90
CRF^b^			
High	1804 (89.5)	212 (10.5)	2016
Median	1607 (84.9)	286 (15.1)	1893
Low	110 (74.8)	37 (25.2)	147
Physical test			
Pass	2468 (87.7)	347 (12.3)	2815
Fail	1006 (84.4)	186 (15.6)	1192

^a^BMI = body mass index and ^b^CRF = cardiorespiratory fitness.

**Table 3:** Risk factors associated with hypertension in binary logistic regression analysis.

Risk factors		*B*	SE	*P*	OR	95% CI for OR	
						Lower	Upper
Test time	2014						
	2005	−.691	.134	.000	.501	.385	.652
	2010	−1.141	.132	.000	.320	.247	.414

Age (yrs)	40–44						
	20–24	−.890	.164	.000	.411	.298	.566
	25–29	−.909	.156	.000	.403	.297	.547
	30–34	−.714	.149	.000	.490	.366	.656
	35–39	−.590	.146	.000	.554	.417	.737

Sex	Women	−.683	.104	.000	.505	.412	.619

Employment classification	Blue collar workers in urban area						
	Blue collar workers in rural area	.553	.165	.001	1.739	1.258	2.403
	White collar workers	.311	.166	.060	1.365	.987	1.888

CRF^b^	High						
	Low	1.070	.225	.000	2.914	1.877	4.526
	Median	.214	.107	.046	1.238	1.004	1.528

BMI^a^	Normal weight						
	Obesity	1.835	.242	.000	6.265	3.896	10.072
	Overweight	.844	.111	.000	2.325	1.870	2.892

	Constant	−.591	.231	.011	.554		

^a^BMI = body mass index and ^b^CRF = cardiorespiratory fitness.
